# Idiopathic intracranial hypertension patients can be effectively managed using an OCT-based telemedicine model

**DOI:** 10.1038/s41433-026-04385-5

**Published:** 2026-03-16

**Authors:** Shai Cohen, Tamir Weiss, Yaron Sela, Anat Bachar Zipori, Meital Ben-Dov, Wahbi Wahbi, Ainat Klein

**Affiliations:** 1 Ophthalmology Devision, Tel Aviv Sourasky Medical Center, Faculty of Medical & Health Sciences, Tel Aviv University, Tel Aviv Israel; 2 Ophthalmology Devision, Moorfields Eye Hospital, University College London Barts and The London School of Medicine and Dentistry City, University of London, London, UK; 3 Sammy Ofer School of Communications, Reichman University, Hertsliya, Israel; 4 Ophthalmology Devision, Columbia University Irving Medical Center, New York, NY USA

**Keywords:** Optic nerve diseases, Tomography

## Abstract

**Background:**

Idiopathic Intracranial Hypertension (IIH) is a syndrome that requires frequent monitoring and long-term follow-up. The shortage of neuro-ophthalmologists, along with the high burden of frequent visits, raise the need to establish a telemedicine model for follow-up and treatment of IIH patients. The aim of this study is to evaluate the feasibility of a Heidelberg Spectralis Optical Coherence Tomography (OCT)-based Telemedicine model for IIH patients.

**Methods:**

Treatment recommendations made by the same physician during real clinic visits, in which the patient was physically examined in the clinic, were compared to those based solely on a simulated remote consult, in which the physician reviewed only the patient’s medical history, OCT Retinal Nerve Fibre Layer (RNFL) scan results and Humphrey Visual Field (HVF) without seeing the patient in person. The agreement rate was evaluated.

**Results:**

Among 62 patient visits included in the study, 54 (87%) were female, with a mean age of 30 (range 6–64). The total agreement rate between two visits type was 87%. The overall kappa measure for the level of agreement between the telemedicine and original decision, was 0.763 (*p* < 0.001), reflecting high agreement. Changes in the OCT RNFL thickness, along with the presence of an enlarged blind spot, tended to result in greater discrepancies between decisions. Conversely, the absence of current medical treatment inclined toward a better agreement between both types of encounters.

**Conclusions:**

In this cohort, simulated remote consult treatment recommendations were predominantly similar to real clinic visit recommendations.

## Introduction

Idiopathic Intracranial Hypertension (IIH), also known as Pseudo Tumor Cerebri (PTC) is a condition in which symptoms and signs of increased intracranial pressure (ICP) present, without evidence of a space-occupying lesion, malignancy, or infection [1, 2]. Most patients are obese women at childbearing age [3]. Although the exact pathophysiology of IIH is unknown, a metabolic syndrome, predominantly obesity and weight gain, seem to play a main rule [4]. The observation of dural sinus stenosis in patients with IIH has given rise to a theory that the condition is driven by a positive feedback cycle [5] including impaired cerebrospinal fluid (CSF) absorption[6–8], increased cerebrospinal fluid (CSF) production, and increased cerebral venous pressure and/or venous stenosis [9]. Patients require frequent monitoring and long-term follow-up due to the risk of vision loss and the chronicity of the disease [10]. Recent consensus guidelines [11, 12] expert opinions [13] and practitioners’ surveys [14] had recommended Optical Coherence Tomography (OCT)-based follow-up protocols.

The high burden of frequent visits, shortage of available neuro-ophthalmologic consultants [15], as well as situations of pandemic contagious disease, raise the need to establish a telemedicine model for follow-up and treatment of IIH patients.

Telemedicine-based visits may require shorter visit times, allowing more visits per day and allow access to neuro-ophthalmological consultation in rural and distant areas and better compliance. A survey by the NANOS conducted in May 2020 reported that the COVID-19 pandemic dramatically increased the use of telehealth by neuro-ophthalmologists. Leong et al. [16] recommend that neuro-ophthalmologists design and implement tele-neuro-ophthalmology protocols for pre-visit, in-visit, and post-visit stages in a practical manner.

Although Telemedicine has proven to be effective in other fields of ophthalmology [17–19], to date, the literature has scarce data regarding the usefulness or safety of telemedicine in neuro-ophthalmology.

Until now, no study methodically evaluated the reliability of a remote telemedicine practice, in managing various neuro-ophthalmic clinical situations and no study examined if those management models suggested and practiced are safe and have similar results to the traditional face-to-face medical encounters.

This present study was designed to evaluate the feasibility of an OCT-based, remote, non-encounter consult model for IIH patients.

## Material and methods

### Acquisition of data

This prospective cohort study was conducted in a single tertiary medical centre (Tel Aviv Medical Center, Israel) on 62 patients with IIH who were regularly followed and treated in our ophthalmology clinic between May 2018 to October 2022.

The study protocol was reviewed and approved by the ethics committees of the participating medical centre in compliance with the tenets of the Declaration of Helsinki, approval no. TLV-0363-20. Informed consent was exempted due to the nature of the study and the use of de identified medical information and ocular finding images.

All participants that were included in the study were diagnosed with IIH according to the modified Dandy criteria [20] and were under regular follow-ups in our clinic. The data collected included age, sex, disease course (time from diagnosis, current treatment regimen and its duration, interval from previous visit), and current symptoms, including typical high ICP related headaches (more severe after the lying down, with gradual improvement in upright position), tinnitus and transient visual obscuration. We also included physical exam findings, including best-corrected visual acuity (BCVA), Humphrey Visual Field (HVF) defects, mean deviation (MD) as per HVF, the presence of relative afferent papillary defect (RAPD), optic disc appearance, and retinal nerve fibre layer (RNFL) appearance and thickness. The RNFL thickness was evaluated by a Heidelberg spectralis optical coherence tomography (HS-OCT), and measured using the standard method of average of one circular scan 3.4 mm from the centre of the optic disc. As previously published the OCT RNFL repeatability is around 5 mm for normal patients, and even higher (8 mm) in abnormal optic nerves as with significant NFL thickening, as in patients with PTC [21]. For this reason, OCT improvement was defined as a decrease in the average thickness of 10 μm or more between the current and previous visit. Deterioration was defined as an increase of 10 μm or more. A change of less than 10 μm was defined as “no significant change.” Finally, although the optic disc couldn’t be evaluated by a dilated fundoscopy in the  simulated remote consult, its appearance was assessed using the OCT’s infra-red images for the presence of optic disc oedema and disc haemorrhages.

### Telemedicine evaluation

To evaluate the utility of Telemedicine evaluation, every physician was presented with data from a previous real clinic visit and was required to make clinical recommendations in a simulated remote consult without examining the patient himself; Telemedicine visits were actually simulated- a mock remote consult visit was set up using the exact same date from a previous real clinic visit. We used only data and visits from an ambulatory follow up of previously diagnosed patients (and not first consult visits). All medical parameters- medical history, VA, VF, OCT RNFL, visual field- all were “copied” from a previous real clinic visit- and presented to the physician now- in the simulated remote consult visit. The data provided included all the medical history of the patients, disease clinical course, and all previous neuro-ophthalmology consult findings and recommendations. They were also presented with the current patient’s complaints/ symptoms and recent HVF and OCT findings. They were not provided with the fundus colour picture nor were they provided with the recommendations made during the actual real clinic visit. They were then asked to make clinical recommendations regarding further treatment; continue the same regimen/increase/decrease the dose or ask for more tests and workup. This was termed “remote consult decision.”

To avoid interobserver disagreement, every patient in simulated remote consult was “evaluated” by the same neuro-ophthalmology specialist who made the original decision in the actual real clinic visit that took place a few months prior. Only visits from at least 12 months ago were used, in order to prevent identifying the patient/case or remembering previous decisions made.

The primary outcome of this study is the degree of agreement between the clinic visit decision and the simulated remote consult decision.

### Statistical analysis

The data were analysed using SPSS version 27. Descriptive statistics were performed using means and standard deviations for the continuous variables and frequencies for the discrete variables. Tests between independent samples were done using the chi-square (${\chi }^{2}$) test for categorical variables and the Mann-Whitney tests for continuous variables. To assess predictors of change in the physician’s decisions between frontal clinic visits and remote consult, a multivariate logistic regression was conducted. Significance was considered for a p-value lower than 5%.

## Results

The cohort comprised 62 patients, with 54 females (87.1%). The average time from initial diagnosis was 46 months (SD = 54.98). The mean age was 30 years (range, 6–64 years), of which 11 (18%) were paediatric patients. Forty patients (65%) were under medical treatment, and 38 (92%) of them were treated with acetazolamide (at an average dose of 1000 mg/d), three of them combined with topiramate mainly for headache control. The other three patients (8%) were treated with furosemide, due to severe side effects of acetazolamide and topiramate, at an average dose of 40 mg/d.

We calculated the agreement between the original clinic visit and simulated remote consult decisions. Overall, the agreement rate between the two visits’ decisions was 87.1%; in 54 cases the physician made the same treatment decision between clinic visit and the simulated remote consult, and in 8 (12.9%) cases the recommendation differed from the original one made in the real clinic visit. The overall Kappa measure for the level of agreement between the telemedicine and original decision, was 0.763 (*p* < 0.001), reflecting high agreement.

The discordance between decisions did not follow a specific rule or trend and varied; in three cases, the simulated remote consult recommendation was to decrease acetazolamide dose in contrast to continuing the same regimen that was recommended in the real clinic visit. In four cases, the simulated remote consult recommendation was to continue the same regimen in contrast to the recommendation to decrease the medication dose in the original clinic and in one case simulated remote consult decision was to decrease treatment while in the original clinic visit the physician recommended to increase the dose.

A comparison of baseline characteristics of patients in these two groups are detailed in Tables 1 and 2. Results show that the groups were similar across all the baseline characteristics. There was also no statistically significant difference in examination indices (i.e., VA, the presence of RAPD, and optic disc characteristics) between the two groups.

**Table 1:** Comparison between groups in demographic and baseline characteristics.

	No difference between decisions		Difference between decisions				
	*N* (%)	M ± SD	*N* (%)	M ± SD	χ^2^	Z	*p*
Sex					0.001		0.971
•Male	7 (13.0%)		1 (12.5%)				
•Female	47 (87.0%)		7 (87.5%)				
Age		29.50 ± 13.62		34.50 ± 13.47		0.95	0.339
Time since diagnosis		49.87 ± 57.10		20.13 ± 28.12		1.78	0.074
Time between visits		4.19 ± 3.17		3.25 ± 2.25		0.63	0.525
Symptoms	23 (42.6%)		5 (62.5%)		1.11		0.291
Headache	28 (51.9%)		4 (50.0%)		0.01		0.922
Tinnitus	14 (25.9%)		3 (37.5%)		0.49		0.493
TVOS	3 (5.6%)		0 (0)		0.46		0.494

*M* mean, *SD* standard deviation, *TVOS* transient visual obscurations.

**Table 2:** Comparison between groups in clinical indices.

	No difference between decisions		Difference between decisions				
	*N* (%)	M ± SD	*N* (%)	M ± SD	χ^2^	Z	*p*
VA RE (6/6)	39 (72.2%)		4 (50.0%)		1.619		0.203
VA LE (6/6)	34 (63.0%)		4 (50.0%)		0.494		0.482
RAPD RE (positive)	1 (1.9%)		0 (0%)		0.151		0.698
RAPD LE (positive)	3 (5.6%)		1 (12.5%)		0.557		0.456
RE Optic Disc - Frisen scale					0.365		0.947
•0	37 (68.5%)		6 (75.0%)				
•1	7 (13.0%)		1 (12.5%)				
•2	8 (14.8%)		1 (12.5%)				
•3	2 (3.7%)		0 (0%)				
RE Optic disc colour					1.560		0.212
•Normal	45 (83.3%)		8 (100.0%)				
•Pale	9 (16.7%)		0 (0%)				
LE Optic Disc - Frisen scale					0.690		0.876
•0	36 (66.7%)		5 (62.5%)				
•1	9 (16.7%)		1 (12.5%)				
•2	8 (14.8%)		2 (25.0%)				
•3	1 (1.9%)		0 (0%)				
LE Optic disc colour					1.361		0.243
•Normal	46 (85.2%)		8 (100.0%)				
•Pale	8 (14.8%)		0 (0%)				
Treatment					5.05		0.042
•None	22 (40.7%)		0 (0%)				
•Medication	32 (59.3%)		8 (100.0%)				
Dosage							
•Stable	25 (46.3%)		1 (12.5%)				
•Increased	11 (20.4%)		3 (37.5%)				
•Decreased	17 (31.5%)		4 (50.0%)				
•Another medication was added	1 (1.9%)		0 (0%)				
Acetazolamid dosage		1043.1 ± 630		1000.0 ± 517		0.761	0.860
Duration of the current treatment		11.36 ± 19.54		4.78 ± 5.86		1.315	0.189

*M* mean, *SD* standard deviation, *VA* visual acuity, *RE* right eye, *LE* left eye, *RAPD* relative afferent pupillary defect.

However, patients without current medical treatment were more likely to receive the same recommendation in both visits type (*p* = 0.042). There was no rule in the discordance between decisions- remote consult decisions were not more conservative /cautious compared to the clinic visit decisions- the “misjudgment” was equivocal between groups.

Clinical parameters including OCT and VF parameters are presented in Table 3. A change of 10 microns or more in OCT RNFL thickness in at least one eye correlated with disagreement between two visits (*p* = 0.044). The presence of an enlarged blind spot was associated with greater likelihood of discordance between two visits recommendations. (For both the right and left eyes, *p* = 0.004 and *p* = 0.048, respectively, as well as calculating for both eyes, *p* < 0.001).

**Table 3:** Comparison between groups in the VF and OCT RNFL scan.

	No difference between decisions		Difference between decisions				
	*N* (%)	M ± SD	*N* (%)	M ± SD	χ^2^	Z	*p*
RE / LE OCT average RNFL compared to previous OCT					6.22		0.044
•No change	76 (70.4%)		10 (52.6%)				
•Improvement	21 (19.4%)		9 (47.4%)				
•Deterioration	11 (10.2%)		0 (0%)				
VF RE MD		−1.87 ± 2.72		−2.16 ± 2.27	0.084		0.933
VF RE enlarged blind spot	3 (5.6%)		3 (37.5%)		8.134		0.004
RE scotoma	4 (7.5%)		0 (0%)		0.646		0.421
VF LE MD		−2.79 ± 4.01		−2.67 ± 1.89	0.963		0.488
VF LE enlarged blind spot	6 (11.1%)		3 (37.5%)		3.910		0.048
LE scotoma	9 (17.0%)		2 (25.0%)		0.302		0.582

*VF* visual filed, *OCT RNFL* optical coherence tomography retinal nerve fibre layer, *M* mean, *SD* standard deviation, *VA* visual acuity, *RE* right eye, *LE* left eye, *MD* mean deviation.

Similarly, the HVF mean deviation and the presence of specific visual field defects (either superior/inferior altitudinal or arcuate defects) did not differ significantly between the groups, with *p*-values of 0.933 and 0.421 for the right eye and 0.488 and 0.582 for the left eye, respectively. To assess the predictors of change in the physicians’ decisions, a logistic multivariate model was applied with the following predictors: age, gender, time since diagnosis, VA RE, VA LE, RE average RNFL Thickness, LE average RNFL Thickness, VF RE enlarged, blind spot, and VF LE enlarged blind spot. The results revealed that no factors significantly affected differences in physicians’ decisions. This suggests that physicians’ recommendations were generally consistent, regardless of whether the visit was a clinic visit or a remote consult.

## Discussion

Due to the shortage of neuro-ophthalmologists [15] and the high burden and long follow-up needs in IIH patients, this study aimed to evaluate telemedicine’s feasibility in IIH patients.

In our study, the agreement rate between clinic visits and simulated remote consults was high, as calculated both by rates of similar decision (87.1%) or by Kappa agreement coefficient (0.76). This high agreement rate demonstrates that telemedicine-based follow-up for IIH patients is feasible and safe, as in most cases there was no difference in management between the two kinds of visits. This is in contrast to Xu et al. [22], who published a review of their experience with telemedicine in neuro-ophthalmology and reported that only in 44% of their patients, the neuro-ophthalmologist was able to provide a clinical decision, whereas for the rest of the cases, further information was needed. It should be noted that their cohort included consults mainly due to vision loss and/or visual field disturbances (35.0%) and optic neuropathies (27.5%), and only 21.3% were due to optic disc oedema from various causes. Their population was different than our current study, in which all patients have been followed due to papilledema secondary to previously diagnosed IIH.

To identify which parameters might influence the disagreement rate, we further analysed the data and found that most cases of discordance occurred in patients demontrating changes and/or positive clinical signs. A statistically significant difference was observed in patients who showed OCT changes of 10 microns or more in at least one eye between visits, as well as in those with an enlarged blind spot on the visual field examination. The enlarged blind spot, which is associated with papilledema [23], and with changes in RNFL thickness, likely prompted physicians to adopt a more cautious approach in treatment management. Stable patients that were not on current ICP-lowering drugs at the time of their visit tended to have more agreement between the two visit types (*p* = 0.042). There was also a trend towards significance to agreement with a longer time since the initial diagnosis (*p* = 0.074). A possible explanation is that these patients were relatively stable and required minimal changes in their medical regimen.

When examining the correlation with the physician providing the consult, it was found that most disagreements were associated with two out of the three physicians involved in the study. The percentage of disagreement was much higher in examiners number 2 and 3 (25% and 37.5%) and much lower in examiner number 1 (7.3%). One explanation for that is that those two physicians were younger than examiner number 1 and had less clinical experience. Notably, the visits selected for review in this study were chosen to be distant from the time the study took place in order to prevent physicians from recalling details that could bias their decision-making. To note, there was no difference in the clinical characteristic of patients between groups. Though the number of patients was not equally distributed among the three examiners; 41 examiner 1 (A.K), 10 examiner 2 (B.M) and 11 examiner 3 (W.W). The groups were too small to elicit statistical significance difference between the groups.

The main limitation of this study is that the simulated telemedicine were actually conducted via a remote review process, a simulated remote consult, with physicians reviewing information and making decisions without real-time interaction through phone or video calls. As a result, the physician did not have the opportunity to hear the patient’s complaints or descriptions of pain and side effects directly, nor could they observe the patient’s facial expressions and overall appearance, including weight. These factors might influence the physician’s decision-making process.

In this study, in the simulated remote consult, physician could review the infra-red images of the optic nerve as captured in the OCT and did not have a colour fundus images of the patient. Though infra-red view dose not enable to appreciate optic nerve colour or hyperaemia, it can detect or exclude peripapillary haemorrhages and outline optic nerve borders. OCT cross-sectional and *en face* imaging can also detect outer retinal changes such as subretinal fluid, peripapillary wrinkles, inner retinal folds, outer retinal folds and creases, and choroidal folds [24], findings that can facilitate physicians making clinical decisions.

Another limitation is that the study included only one visit per patient, and we don’t have long-term follow up of patients being treated by one method or the other, so long-term impact on vision couldn’t be evaluated. Furthermore, since visits selected for review in this study were chosen to be distant from the time the study took place, as detailed above, it is possible that clinician’s approach has changed during the years, becoming less or more aggressive, and that this could explain the differences between recommendations. Still, no trend was among examiners, except for the rate of discordance between decisions.

Another possible way to further strengthen this study’s results could have been to have two simulated remote consults for each patient, and then compare clinic visits to remote consult and also one remote consult to the second remote consult. Unfortunately, this was not done as part of the study design.

Using clinical data, OCT scans and VF from routine in patient visits that took place remotely and not as part a strict study protocol, could be seen as a limitation. Still, our medical centre has high qualified medical technicians who use strict protocol for neuro ophthalmic patients, as well as we only included high quality OCT images as regard to signal strength, and images were also examined by the physicians to ensure correct segmentation, further studies in this area should embed a central reading centre and follow all APOSTLE guidelines for quality OCT capture and OSCAR-B guidelines for reporting [25, 26].

This study utilised a simulated remote consult, demonstrating general agreement with the real clinic visit. We believe that incorporating virtual visits, which allow for real-time interaction with the patient through video, could further enhance the potential of telemedicine. By more closely resembling in-person consultations, virtual/telemedicine visits may reduce discrepancies and improve agreement rates between clinical decisions. Additionally, given the shortage of practicing neuro-ophthalmologists, this approach is particularly valuable and necessary.

## Conclusions

In this study, remote consult-guided treatment and follow-up were found to result in similar treatment decisions for IIH patients compared to a face-to-face clinic visit. According to our results, it seems that when circumferences make clinic visits impossible, remote OCT-based telemedicine can be a suitable substitute. Caution is warranted in patients with ongoing disease activity.

## Summary

### What was known before

- There are no previous studies on telemedicine in IIH patients.

### What this study adds

- In this study, remote consult-guided treatment and follow-up were found to result in similar treatment decisions for IIH patients compared to a face-to-face clinic visit.

## Supplementary information

REPORTING CHECKLIST

## Data Availability

The data that support the findings of this study are not openly available due to reasons of sensitivity and are available from the corresponding author upon reasonable request. Data are located in controlled-access data storage at Tel Aviv Sourasky Medical Center.
